# Successful delivery of subretinal aflibercept (new surgical technique) for the treatment of submacular hemorrhage in idiopathic polypoidal choroidal vasculopathy

**DOI:** 10.1093/jscr/rjab358

**Published:** 2021-08-16

**Authors:** K V Chalam, Suzie Gasparian

**Affiliations:** Department of Ophthalmology, Loma Linda University Medical School, Loma Linda, CA, USA; Department of Ophthalmology, Loma Linda University Medical School, Loma Linda, CA, USA

## Abstract

Submacular hemorrhage (SMH) is often a result of trauma, wet age-related macular degeneration or IPCV and frequently leads to blindness secondary to extreme toxicity of hemoglobin products on photoreceptors. We describe a new technique of subretinal aflibercept injection during pars plana vitrectomy for the treatment of SMH in idiopathic polypoidal choroidal vasculopathy (IPCV). A 55-old male presented with sudden loss of vision (HM) secondary to massive subretinal hemorrhage associated with IPCV. Subretinal injection of aflibercept with a 25 g/42 g cannula coupled to the viscous fluid control unit of a standard vitrectomy system was performed during parsplana vitrectomy. Controlled injection of aflibercept intra-operatively has resulted in a resolution of SMH (confirmed with OCT and ICG). Visual acuity improved from HM to 20/20. This combined approach delivered anti-VEGF agent to target tissue in controlled fashion with the assistance of VFC system (similar to gene therapy) and restored full vision with resolution of SMH.

## INTRODUCTION

Submacular hemorrhage (SMH) is a major vision-threatening complication of a variety of retinal diseases, including wet age-related macular degeneration (AMD) and Idiopathic polypoidal choroidal vasculopathy (IPCV) [1–3]. Subretinal blood induces irreversible functional and anatomical damage to photoreceptors (from iron toxicity) and often leads to blindness [4].

Treatment of SMH remains a difficult challenge [5]. Currently, combination of vitrectomy with intra-vitreal or subretinal *t*-PA with or without gas and intravitreal anti-VEGF therapy is utilized [1–3, 5, 6]. Alternatively, less invasive treatment options include intravitreal injections of gas, *t*-PA and/or anti-VEGF agents [2, 7].

In this report, we describe a new surgical technique for efficient and controlled delivery of an anti-VEGF agent (aflibercept) into subretinal space with the aid of a 25 g/42 g cannula coupled to the viscous fluid control (VFC) unit of a standard vitrectomy system. We believe that the delivery of biologic agent (aflibercept) into subretinal space resolved the underlying pathology and limited the toxic effect of hemoglobin products on photoreceptors through dilution of hemorrhage.

## CASE REPORT

A 55-year-old Caucasian male presented with sudden-onset of loss of vision in his left eye. Best-corrected visual acuity (BCVA) was 20/30 in the right eye (OD) and hand movements (HM) in the left eye (OS). Slit-lamp examination revealed nuclear sclerotic cataracts in both eyes.

Fundus examination of the left eye revealed a large dense subretinal hemorrhage, ~12 disc-diameters in size, covering the entire posterior pole with evidence of fresh subretinal hemorrhage along with multiple polypoidal lesions (Fig. 2A). Fundoscopy of the right eye was normal. Optical Coherence Tomography (OCT; Heidelberg Engineering Inc., Heidelberg, Germany) of the right eye was normal. It could not be performed in left eye due to large subretinal hematoma.

### Surgical technique

**Figure 1:**
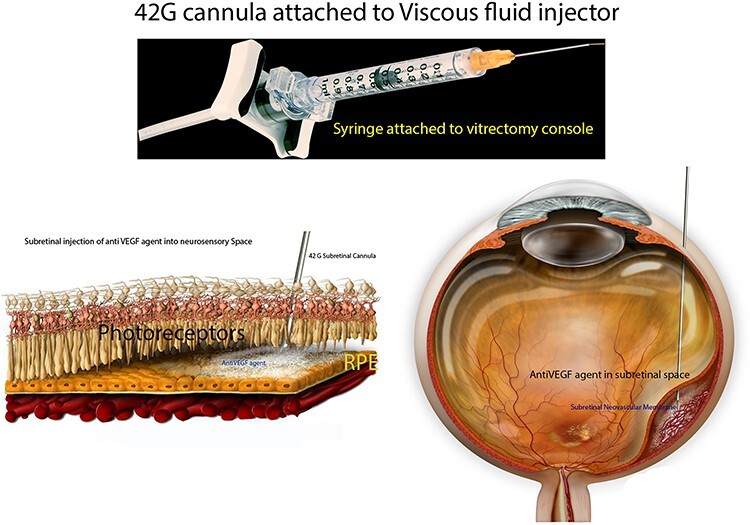
Preparation of the subretinal agent delivery system. (**A**)Prepared 1-mL syringe coupled with a 42 g/25 g cannula connected to the VFC unit of the vitrectomy device. (**B**) Histologic schematic of delivery of anti VEGF agent into subretinal space. (**C**) Schematic of delivery of anti-VEGF agent during vitreoretinal surgery.

About 0.05 cc (2 mg; Eylea; Bayer, Basel, Switzerland) of filterd aflibercept was placed in a custom 1 cc syringe (Medone; Tampa, FL) prior to connection to the VFC unit of a standard vitrectomy (Constellation; Alcon Laboratories, Inc., Fort Worth, TX) system; 25 g/42 g cannula was attached to the 1-mL syringe of the VFC unit (Fig. 1).

Standard 3-port, 23-gauge pars plana vitrectomy was then performed. After completion of core vitrectomy and induction of posterior vitreous detachment, the superotemporal aspect of the macula (about 1 disc-diameter superior to the superior edge of the subretinal hemorrhage) was penetrated with the 25 g/42 g cannula (Fig. 1C). Cannula insertion is facilitated by engaging the retina at a beveled 45–60° angle. VFC was activated with the foot pedal (10 PSI) and aflibercept was delivered into the subretinal space (with the aid of intraoperative OCT); dilution of subretinal hemorrhage was noted. Complete gas-fluid exchange was performed and sclerotomies were closed.

### Post-operative course

**Figure 2:**
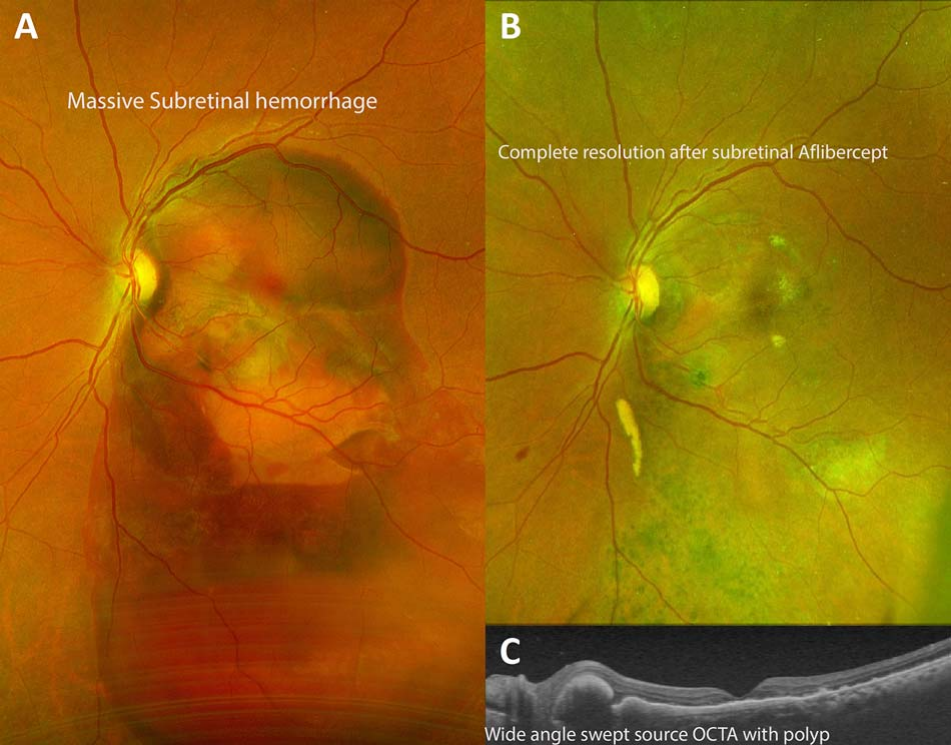
Demonstration of successful resolution of SMH in IPCV by Optos ultra-wide field fundus photography (Optos, Marlborough, MA) and Heidelberg Spectralis OCT (Heidelberg Engineering Inc., Heidelberg, Germany). (**A**) Fundus photograph at initial visit demonstrating a massive dense subretinal hemorrhage covering entire posterior pole of right eye. (**B**) Fundus photograph 3 months after pars plana vitrectomy with delivery of subretinal aflibercept demonstrating complete resolution of SMH. (**C**) Swept source OCT of the macula 1 month post-operatively demonstrating return of near-normal macular contour with polyps associated with idiopathic polypoidal choroidopathy.

**Figure 3:**
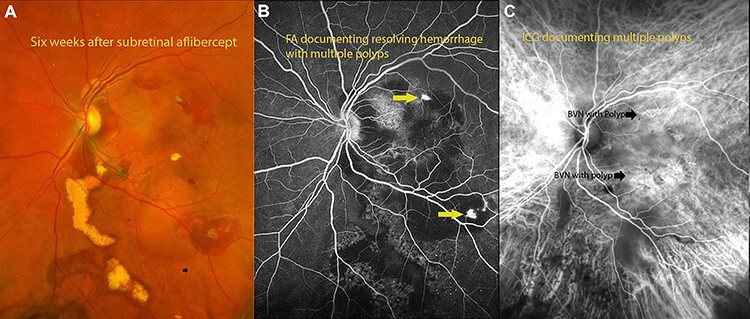
(**A**) fundus photograph 6 weeks after surgery documenting resolving hemorrhage with residual exudates from polyps. (**B**) Flourescein angiography documenting polyps with residual hemorrhage. (**C**) Indocyanin angiography demonstrating areas of polyps with BVNs in the areas of polyps.

**Figure 4:**
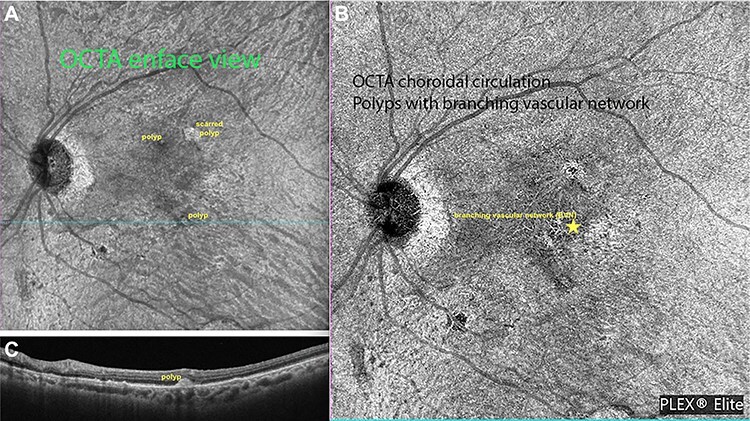
Ziess Plexelite OCT angiography images of IPCV before and after intervention. (**A**) OCT angiography enface image of a polyp with subretinal hemorrhage before surgery. (**B**) OCT angiography enface image of resolved hemorrhage with regressed polyp.

Complete resolution of subretinal hemorrhage along with anatomical improvement was confirmed by ultra-wide field fundus photography and wide field OCT imaging (Fig. 2C and D). BCVA was 20/200 on first post-operative day and improved to 20/20 at 3 month follow-up visit. FA and ICGA revealed persistent polypoidal lesions in the macula during the early phases of the angiogram (Fig. 3C). Complete resolution of the hemorrhagic manifestations attributable to the polypoidal lesions and scarring of few polyps was noted (Fig. 2B). OCT angiography (OCT-A) demonstrated active and inactive polyps with scarring along with branching vascular network (BVN) on corresponding B-scan (Fig. 4).

## DISCUSSION

SMH is a relatively common and challenging complication of IPCV with poor prognosis [3, 8]. We report the first case of successful displacement and resolution of SMH using a combined vitrectomy and subretinal anti-VEGF delivery technique in IPCV.

Subretinal hemorrhage typically arises from the choroidal or retinal circulation and is an accumulation of blood between the neurosensory retina and RPE. Multiple techniques for SMH displacement have been proposed, but data pertaining to management of subretinal hemorrhage in IPCV remains limited. Intra-vitreal or subretinal *t*-PA is employed in the management of subretinal hematoma in conjunction with intravitreal anti-VEGF agents (many in the setting of neovascular AMD) in various studies [1–3, 5, 7–10]. One study reported successful SMH displacement with subretinal application of *t*-PA followed by intra-vitreal injection in neovascular AMD in 35 of 41 eyes [6]. Kitahashi *et al*. compared pneumatic displacement (PD) with intravitreal bevacizumab to PD alone for the treatment of SMH in IPCV and found complete displacement from under the fovea in 86.4% in PD with intra-vitreal bevacizumab versus 50% in the PD only group [9].

We modified previously reported surgical procedures and combined pars plana vitrectomy with delivery of aflibercept into the submacular space (site of pathology) in efficient and controlled manner with the aid of VFC system to control neovascular process as well as dilute subretinal blood. Unlike with intra-vitreal administration (where drug has to penetrate retina to reach subretinal space), high concentration of anti VEGF agent is delivered to target tissue with this modified approach. VFC unit of the vitrectomy system provided a controlled, efficacious anti-VEGF delivery into the submacular space, similar to techniques employed in gene therapy protocols. Aflibercept has proven successful in IPCV for management of recurrence of polyps. Once bleeding occurs in IPCV, re-bleeds are more likely to occur within a short period [10]. Our patient had no recurrence of hemorrhage during follow-up.

In view of extreme iron toxicity to photoreceptors, timing is a crucial factor in adequate displacement of SMH. Our patients’ visual acuity considerably improved with prompt surgical intervention, which further highlights the association between duration of SMH displacement and visual outcomes. Independent factors affecting BCVA 6 months after treatment included pre-treatment BCVA, pre-treatment central ellipsoid zone, pre-treatment central PED thickness and post-treatment central PED thickness [9], which further substantiates our hypothesis.

Our result suggest that vitrectomy with subretinal injection of aflibercept is a safe and effective treatment option for resolution of SMH associated with IPCV.

In conclusion, we report a new, safe, effective and affordable method of subretinal delivery of aflibercept with a 25 g/42 g cannula coupled to the VFC unit of a standard vitrectomy system in the management of SMH in IPCV. This novel surgical approach facilitated successful delivery of therapeutic agent into subretinal space with complete resolution of the SMH and good recovery of visual acuity. We believe that this method may also be used in instances of SMH in monocular patients with wet macular degeneration.
